# Stress Triggers Flare of Inflammatory Bowel Disease in Children and Adults

**DOI:** 10.3389/fped.2019.00432

**Published:** 2019-10-24

**Authors:** Yue Sun, Lu Li, Runxiang Xie, Bangmao Wang, Kui Jiang, Hailong Cao

**Affiliations:** Department of Gastroenterology and Hepatology, General Hospital, Tianjin Medical University, Tianjin, China

**Keywords:** inflammatory bowel disease, stress, pediatrics, gut microbiota, brain-gut axis, treatment

## Abstract

Inflammatory bowel disease (IBD) is an idiopathic inflammatory disease characterized by chronic and relapsing manifestations. It is noteworthy that the prevalence of IBD is gradually increasing in both children and adults. Currently, the pathogenesis of IBD remains to be completely elucidated. IBD is believed to occur through interactions among genetics, environmental factors, and the gut microbiota. However, the relapsing and remitting course of IBD underlines the importance of other modifiers, such as psychological stress. Growing evidence from clinical and experimental studies suggests that stress acts as a promoting or relapsing factor for IBD. Importantly, recent studies have reported an increasing incidence of anxiety or depression in both children and adults with IBD. In this article, we review the mechanisms by which stress affects IBD, such as via impaired intestinal barrier function, disturbance of the gut microbiota, intestinal dysmotility, and immune and neuroendocrine dysfunction. With regard to both children and adults, we provide recent evidence to describe how stress can affect IBD at various stages. Furthermore, we emphasize the importance of mental healing and discuss the value of approaches targeting stress in clinical management to develop enhanced strategies for the prevention and treatment of IBD.

## Introduction

Inflammatory bowel disease (IBD), which includes ulcerative colitis (UC), and Crohn's disease (CD), is a chronic, relapsing, and remittent intestinal inflammatory disorder (1) affecting millions of people worldwide (2). Notably, IBD is gradually becoming a global disease with rapidly increasing incidence in emerging industrial countries in the twenty-first century (3). Although IBD can occur at any age, ~25% of patients are diagnosed with IBD before 20 years of age (4). The incidence of IBD in children varies among different countries, but the overall trend is increasing globally. The incidence is about 0.5–23/1,00,000 for IBD, 0.1 to 13.9/1,00,000 for CD, 0.3 to 15.0/1,00,000 for UC (3, 5–7). In addition to the common gastrointestinal symptoms (abdominal pain, diarrhea, hematochezia, and weight loss) similar to those of adults, children may present with unique manifestations, including poor growth and delayed puberty (8). IBD is considered to be an immune-mediated intestinal disorder, resulting from complex interactions among genetics, environmental factors, and gut microbiota (9). Various factors, such as genetic transmission, intestinal immune disruption, gut microbiota disturbance, diet, infection, lifestyle, psychological stress, sleep disorders, smoking, and early life exposure to antibiotics, have been found to influence the progress of IBD on the basis of studies in recent decades (10, 11). However, the exact pathophysiological mechanism of IBD remains to be inadequately understood. Its complex and multifactorial pathogenesis, severity of symptoms, uncertainty of the condition and prognosis, and adverse reactions to medication and cancer risk bring multiple challenges to the cure of IBD. Owing to these challenges, patients' quality of life may be significantly affected, particularly by increasing psychosocial burdens and inducing psychological disorders. For children and adolescents, IBD can even threaten the healthy psychosocial development.

Stress may cause abnormalities of behavior and/or mentality, such as anxiety and depression, and also influence the function of visceral organs, especially the digestive system. Psychological comorbidities, especially depression, have similar pathophysiological mechanisms to IBD. Pro-inflammatory cytokines and plasma acute phase protein C increased in depression patients (12). Elevated levels of malondialdehyde, a fatty acid peroxide, in the serum of depressed patients suggests that mental disorders may be associated with oxidation and oxidative stress (13). Meanwhile, autoimmune changes and bacterial translocations have also been observed in depression (14, 15). Thus, depression and IBD share a common pathway that seems to explain the interaction between the two diseases. In recent years, a growing number of studies have indicated that the prevalence of mental disorders in both children and adults with IBD is higher than that of healthy people (16). Significant progress has been made in elucidating the pathophysiological mechanisms of IBD, which indicates that stress is closely correlated with IBD. Accumulating evidence suggests that there is a bidirectional influence between IBD and stress. The underlying mechanisms consist of immune dysfunction, intestinal microbiota disturbance, impaired intestinal barrier function, and neuroendocrine system alterations (17). In addition, whenever stress occurs, whether in early life or adulthood, the development of IBD can be affected to some extent.

This paper provides an overview of recent literature focusing on the connection between stress and IBD in children and adults. Both experimental and clinical evidence illustrating the importance of stress in the pathogenesis of IBD is presented in this review. Finally, insights into comprehensive approaches for managing IBD and potential therapeutic implications of psychological interventions are provided.

## Stress: Pathways and Pathophysiology

More than 80 years ago, Hungarian endocrinologist Hans Hugo Bruno Selye first defined the medical term “stress” as the physiological adaptive responses of organisms to adverse threats (stressors), which are endogenous or exogenous, psychological or physical, real or perceived (18). To maintain homeostasis under threat, organisms have evolved an extremely complex system, called the stress system, which involves physiological and behavioral adaptations via appropriate central and peripheral neuroendocrine responses. When exposed to long-term or severe stress, the organisms may reach a state called cacostasis, in which many vital physiological functions are impaired, and may develop many acute and chronic diseases (19). Stress-induced disorders occur in multiple systems throughout the body, among which the gastrointestinal tract is a sensitive system.

**Figure 1:**
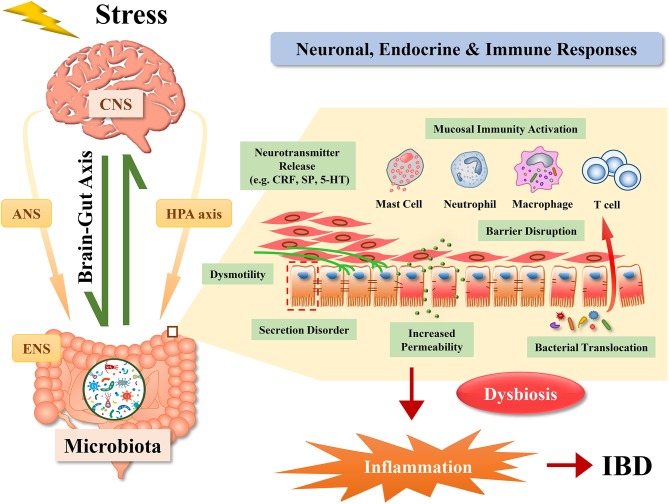
Pathways by which stress affects inflammatory bowel disease. Effects of stress on the gut involve comprehensive reactions among neuronal, endocrine, and immune systems. Stress induces the activation of the brain-gut axis, the hypothalamic-pituitary-adrenal axis (HPA axis), the autonomic nervous system (ANS) and the enteric nervous system (ENS), contributing to the development of inflammatory bowel disease (IBD) via dysbiosis, alterations of secretion and mobility, disruption of the intestinal barrier and the release of inflammatory mediators. CNS, central nervous system; CRF, corticotrophin releasing factor; SP, substance P; 5-HT, 5-hydroxytryptamine.

When the brain receives stress input, multiple pathways containing the autonomic nervous system and hypothalamic-pituitary-adrenal axis (HPA axis) are activated (20). Stress from different sources results in modifications of the brain-gut axis, which eventually leads to the progression of a wide range of gastrointestinal disorders. The frequently involved diseases include IBD, irritable bowel syndrome (IBS), peptic ulcers, food antigen allergic reactions, and gastroesophageal reflux disease. The potential mechanisms are summarized in the following sections (Figure 1).

### HPA Axis

Corticotrophin-releasing factor (CRF) is considered to be a pivotal component in the HPA axis. It is produced by central and peripheral tissues in response to stress. CRF plays an important role in translating stimuli into physical responses in the brain (21). Stress directly activates the hypothalamus (mainly paraventricular nucleus of the hypothalamus system) to release CRF, inducing the anterior pituitary gland to secrete adrenocorticotrophic hormone, which further stimulates the adrenal cortex to secrete cortisol. Ultimately, cortisol acts on all tissues in the body via the circulation of blood. CRF receptors, as well as their ligands, which can be modulated by stress, are expressed in the gut as well as in the brain (22).

CRF acts on enteric peristalsis, secretion, and the mucosal barrier, playing a role in functional and organic disorders, such as IBS and IBD (23). In experimental animal studies, CRF has the opposite effect on upper and lower digestive transit, such as gastric emptying inhibition, reduction of small intestinal transit and increase of colonic transit and defecation (24, 25). In addition, CRF can induce mast cell degranulation and increase mucosal permeability (26), which is the key mechanism of intestinal disorders. Blocking CRF restrains the development of IBD by inhibiting mast cell degranulation and reducing tumor necrosis factor-alpha (TNF-α) and protease production (27).

The results from clinical studies are consistent with the above findings. In one study, healthy volunteers were asked to give a public speech to induce psychological pressure. The pressure induced by public speaking enhanced the permeability of the gut only in subjects with significantly elevated cortisol levels, suggesting that HPA axis activation is affected. Furthermore, peripheral injections of CRF were administered to reproduce stress-induced disorder. The results showed that exogenous CRF, as well as psychological stress, can increase the ratio of urine excretion rates of milk fructose and mannitol, showing signs of increased permeability in the small intestine. Furthermore, the mechanism appears to rely on intestinal mast cells because it can be abrogated by a mast cell stabilizer (28).

During the stress-induced changes of the gut, effectors cells, including mast cells (28), neutrophils (29) and lymphocytes, as well as pro-inflammatory cytokines, are placed in a pivotal position. Mast cells play an important role in the transmission of stress signals to the gut. Animal experiments have demonstrated that stress can damage the gut barrier function in a mast cell-dependent manner, which may facilitate the development of IBD (30, 31). In wild-type rats, chronic stress can induce intestinal barrier dysfunction, inflammatory cell infiltration, ultrastructural changes in epithelial cells, and mast cell proliferation and activation. In contrast, the intestinal epithelial function and morphology are not damaged in mast cell-deficient rats, and there is no evidence of inflammatory cell infiltration, which highlights the regulatory role played by mast cells (32).

### The Autonomic Nervous System

The sympathetic and parasympathetic autonomic nervous systems serve the entire gastrointestinal tract and are closely connected with the enteric nervous system (ENS). Together, these systems govern secretion, motility, sphincter control, and microcirculation in the gut (33). Under stress conditions, the ENS produces large neuropeptides, which in turn affect intestinal immunity and inflammation. Geboes et al. found that there are mixed abnormalities in all CD and UC patients for different cell types of the ENS (34). Another study showed that patients with UC have markedly lower autonomic functions in comparison to those with CD and healthy controls (35).

Stress can activate the sympathetic autonomic system, leading to increased production of major adrenal medulla hormones, mainly catecholamines, such as epinephrine and norepinephrine. Catecholamines mediate increases of central and peripheral inflammatory cytokines and activation of the inflammatory nuclear factor κB signaling pathway in response to stress (36). In addition, the vagus nerve, which has anti-inflammatory effects, is inhibited by stress, leading to an increased systemic inflammatory response to endotoxin and intestinal inflammation (37, 38).

In addition, changes in tissue levels of neurotransmitters have been demonstrated in patients with IBD. Stress can also affect the follicle-associated epithelial barrier via vasoactive intestinal polypeptide (VIP) and its receptor on mucosal mast cells. These findings highlight an important effect of VIP-bacterial-epithelial interactions on regulating intestinal barrier function (39). In a mouse model of chronic restraint stress, substance P (SP) and its receptors enhanced CRH expression and release in eosinophils, resulting in epithelial barrier dysfunction mediated by mast cells (40). Another study revealed that water avoidance stress (WAS)-induced colonic hypermotility is probably dependent on the upregulation of the neurokinin-1 receptor (NK1R) in the colon and increased serum SP levels, suggesting a potential mechanism for diarrhea in IBD patients with anxiety or depression (41).

### The Microbiota Brain-Gut Axis and the Immune System

The effects of the gut microbiota on IBD have attracted much attention in the last decade. Microbiota communicates with the brain-gut axis through mucosal cells, immune cell, and neural endings (42). Published data from animal and clinical studies indicate that stress causes dysbiosis. Stress-induced dysbiosis is characterized by a decrease in the abundance of Lactobacillus and aggravated bacterial translocation. Notably, reduced *Lactobacillus* abundance contributes to opportunistic infections of *Campylobacter jejuni* and *Shigella flexneri* in monkeys (43). The gut microbiota of male mice exposed to chronic social defeat is characterized by reduced richness and diversity. The predicted functional profile shows reduced functional diversity. In particular, the lower prevalence of pathways involved in the synthesis and metabolism of short-chain fatty acids and neurotransmitter precursors has been described (44). A study showed that exposure to stress inhibits the NOD-like receptor, pyrin domain containing (NLRP)-6 inflammasome, altering the constitution of the gut flora, thus leading to inflammation of the intestine. Interestingly, transmissible intestinal inflammation, accompanied by upregulated CRF and reduced NLRP6, was observed after the mice were cohoused (45).

Stress can also break the established tolerance and augments immune responses in chronic intestinal inflammation. Increased intestinal permeability caused by stress allows microbiota to cross the gut epithelial barrier to trigger the mucosal immune reactions (42) and then transfer to secondary lymphoid organs (46) to activate the innate immune system. A recent study based on a dextran sulfate sodium (DSS)-induced colitis model provided evidence that chronic stress increases sensitivity to colitis via dysbiosis and immune system dysfunction. Under chronic stress conditions, the colonic lamina propria showed B cell, neutrophil, and pro-inflammatory ly6C^hi^ macrophage infiltration. Mesenteric lymph node (MLN) changes were also discovered with a significant change in the proportion of MLN-associated immune cells. The results of this study further showed marked activation of IL-6/STAT3 signaling in response to stress. Interestingly, the detrimental effects of stress were not terminated in *IL-6*^−/−^ mice, indicating that the hyperinflammatory response is not the real culprit. In contrast, when the intestinal microbiota was shared by cohousing or was destroyed by antibiotics, the severity of DSS-induced colitis was indistinguishable between the stressed and control groups, unequivocally suggesting that the gut microbiota is responsible for the deleterious effects of stress. In general, stress disturbs the gut microbiota, triggers immune system dysfunction and facilitates DSS-induced colitis (47). A novel phenomenon has been revealed, showing that stress restrains the suppressive action of intestinal regulatory T cells (Tregs), instead of changing their quantity. It was found that prolactin, a stress-related mediator, can transform the phenotypes of intestinal Tregs, thus contributing to intestinal inflammation (48).

It has been shown that stress-induced flora disturbance has a vital impact on IBD by influencing host-microbiota crosstalk and regulating the neuro-immune-endocrine system (49–51). There is a complex network among the gut microbial landscape, immune system and nervous system. Microbiota-targeted therapies have been highlighted as a novel approach to treat systemic inflammation diseases, such as IBD, multiple sclerosis, systemic inflammatory arthritis, and asthma (52). Generalized microbiota-targeted therapies include antibiotics, antibacterial conjugate vaccines, probiotics, fecal microbiota transplants (FMTs) and other interventions that alter the community composition (53). In conclusion, these therapies might be beneficial to both physical and psychological recovery in IBD patients.

## Interaction Between Stress and IBD

In the 1950s, IBD was considered a psychosomatic disorder (54), and previous studies have demonstrated a close association between IBD and stress. Specifically, IBD patients are often exposed to stress, which induces mood swings or even leads to mental complications. Meanwhile, increased emotional disorders can exacerbate symptoms such as abdominal pain, and can enhance the severity of IBD in turn.

### Prevalence of Psychological Comorbidities in IBD

#### Psychological Comorbidities in Adult IBD

Most clinical studies have shown that mood disorders are associated with an increased risk of a variety of chronic diseases, such as IBD, arthritis, asthma, and diabetes mellitus (55, 56). In an IBD cohort, patients exhibited a high incidence of psychological distress and comorbidities, including depression, anxiety disorders, and bipolar disorder (57). Research from Canada examined the prevalence of depression in two typical surveys in a large sample. Statistical data indicated that the 12-month depression incidence rates of people with IBD and similar intestinal disorders in the survey mentioned above were 14.7 and 16.3%, respectively. Furthermore, IBD patients showed a three-fold higher incidence of depression than healthy people (58). In the National Health and Nutrition Examination Survey (NHANES) of Americans, the relationship between IBD and depression was examined. In this big data study, IBD hallmarked by chronic and recurrent disease, was found to act as an independent risk factor for depression (59). The Canadian Community Health Survey in 2012 reported that IBD is strongly associated with generalized anxiety disorder. Generalized anxiety disorder was identified by the WHO-CIDI lifetime criteria. The results revealed that IBD patients were prone to generalized anxiety with a two-fold increased incidence (60). Neuendorf et al. screened 171 articles, including a total of 158,371 participants, to conduct a comprehensive systematic review. The findings showed that 35% of IBD patients develop anxiety symptoms and 21% develop anxiety disorders; 22% of IBD patients develop depression symptoms; and 15% develop a depressive disorder. Furthermore, this study pointed out that this condition is more prevalent during the active period of the disease (61).

With the objective of exploring the bidirectional relationship, Sexton et al. assessed symptom activity, intestinal inflammation, and perceived stress using the Manitoba IBD Index, fecal calprotectin in the stool, and Cohen's Perceived Stress Scale at months 0, 3, and 6. Perceived stress at month 0 was found to be positively correlated with disease activity at months 3 and 6 in both UC and CD. Nevertheless, no correlation between intestinal inflammation, evaluated by fecal calprotectin and perceived stress, was found (62).

A total of 403,665 patients with depression and 5323,986 people without a history of depression were followed up for an average of 6.7 years. A total of 0.05% of the depression cohort developed CD, while 0.03% of individuals in the non-depression cohort developed CD. Furthermore, 0.13% of patients in the depression cohort developed UC, and only 0.09% of individuals in the non-depression cohort developed UC. Compared with the non-depression cohort, the unadjusted hazards of CD and UC in the depression cohort increased by 67 and 41%, respectively. After adjusting for various confounding factors, the risk of developing IBD remained significantly increased in the depression cohort (63).

#### Psychological Comorbidities in Childhood IBD

Despite the limitation of inclusion age criteria for adolescent subjects, the phenomenon that adolescents with IBD have a higher prevalence of anxiety and depression symptoms can still be concluded (64, 65). According to parental reports, emotional problems, including anxious/depressed mood and withdrawn/depressed mood, appear to be more common in adolescents with IBD than National Health and Nutrition Examination in population-based controls. Both parental and self-reported psychosocial symptoms are related to the increased severity of self-perceived IBD symptoms (66). The incidence of mental illnesses, especially depression, among young people with IBD is increasing (67). In a prospective study of 121 patients with IBD aged 16–21 years, 55% reported increased anxiety/depression symptoms and 83% had a reduced quality of life compared with the baseline (68). A study including 374 IBD patients from the Netherlands found elevated symptoms of psychological comorbidities in both adolescents (10–17 years) and young adults (18–25 years), but there was no difference (64). A Swedish study found that IBD children who were identified with younger than 18 years had a three-fold increased hazard ratio for death in adulthood compared to children in the general population. The highest estimated risk of overall mortality was higher in UC patients than in CD patients (69).

#### Animal Models to Assess Psychological Impact of IBD

Similar phenomena have been observed in animal experiments. Depressive- and anxiety-like behaviors were found in mice with dinitrobenzene sulfonic acid (DNBS)-induced colitis. Upregulated expression of inflammatory genes and mitochondrial dysfunction in the hippocampus might be responsible for the abnormal mouse behaviors (70). Recent studies have shown that mice with chronic colitis exhibit increased anxiety-related behaviors in open-field and acoustic stress tests, accompanied by visceral hypersensitivity and low levels of intestinal inflammation (71).

Overall, IBD patients are more prone to developing emotional disorders than the general population. In addition, depression and anxiety have adverse effects on the course of the disease. Psychological comorbidity and IBD seem to fall into a vicious circle.

### The Impact of Stress on IBD

Life always includes stresses which change over time. In adulthood, stress mainly originates from family, work, economic status, and major life-threatening events. Early life and childhood exposure to antibiotics, vaccination, diet, smoke, and psychosocial stress seems to lead to a long-term adverse influence throughout life. The stressors of the above different periods may increase adulthood susceptibility to diabetes, cardiovascular disease, autoimmune disease, stroke, and certain cancers (72–74).

#### Stress and the Risk of IBD Onset

A Manitoba IBD cohort study in Canada ascertained the first onset of psychotic symptoms via a structured diagnostic interview. The report showed that approximately two-thirds of patients who had both anxiety disorder and IBD actually developed psychiatric symptoms predating the IBD diagnosis by over 2 years. This more than 2-year time interval for diagnosis was also present in more than half of IBD patients with mood disorders. Moreover, IBD patients with lifelong anxiety or mood disorders displayed an earlier onset of IBD symptoms than those without the above disorders, and there was a tendency for an early diagnosis of IBD (75). These results reminded us of the potential interactions between IBD and psychiatric diseases. It is possible that the existence of these psychiatric illnesses may increase the susceptibility of individuals to IBD.

Recent studies have focused on the long-term effects of early life adversity on the immune system, including impaired cellular immunity, increased inflammation, and accelerated immunosenescence (76, 77). An animal experiment showed that early- life stress results in an altered microbiota and increased visceral sensation and psychiatric illnesses (78). A recent study found that nerve growth factor (NGF)-mediated tropomyosin receptor kinase A (TrkA) signaling mediates bowel dysfunctions that resemble IBS induced by neonatal maternal separation (79). Moreover, there are sex differences in the effects of early life adversity on gut microbiota and emotional behaviors (80). Parental separation in childhood can lead to psychological distress in adulthood to varying degrees. The adverse impact caused by this abnormal family pattern contributes to the development of IBD in adulthood (81). Researchers analyzed the relationship between the annual rhythm of IBD symptom onset and academic semesters in children. The results showed that academic stress may facilitate disease onset in pediatric IBD (82).

In animal models, there seem to be different views as to whether early stress increases the incidence of IBD, which may be related to different patterns and periods of stress (83–85).

Animals exposed to WAS developed acute small intestinal inflammation as evaluated by histological scores in an experimental study. Leukocytic infiltration, intestinal hyperpermeability, increased serum TNF-α, and upregulated IL-17 and IL-6 expression in mucosa have also been discovered during stress (45). In addition, acoustic stress has been found to cause severe enteritis in the healthy intestinal tract (86). Chronic stress can cause excessive growth of pro-inflammatory bacteria and thus induce increased susceptibility to colitis in subjects after fecal microbiota transplant. Stress is known to cause low-grade intestinal inflammation via increased bacterial translocation and the production of poisons (87).

#### Effect of Stress on IBD Course

Stress causes wide-ranging effects on patients with IBD, especially the recurrence and aggravation of the disease. Some studies have shown that high perceived stress has a bearing on the frequency of symptomatic flares (88). A systematic review of 15 high-quality studies arrived at the conclusion that emotions are associated with abdominal pain symptoms in IBD patients. Among IBD patients, depression, anxiety, and perceived stress are common emotional disorders (89). Although the symptoms become distinctly intensified, IBD activity evaluated by the fecal calprotectin level may not be apparent during perceived stress (90). In contrast, a German cross-sectional study including 1,032 IBD patients, revealed that relevant reported depressive symptoms correlate with increased rates of disease activity (91). In addition to exacerbating symptoms, stress can also lead to relapse in IBD patients (92). Moreover, in a prospective longitudinal study, 60 patients with quiescent IBD were followed-up for up to 18 months. The baseline depression score was found to be connected with the first recrudescence time. In particular, patients with anxiety appeared to have an increased recurrence frequency (93). A multicenter cohort study in Germany found that patients with CD were more likely to be affected by psychological disorders than those with UC or controls. Compared with the healthy controls, both UC and CD patients scored higher on psychological disorders and maladaptive stress coping tests during the active phase. Interestingly, UC patients in remission were minimally affected by psychological disorders, while CD patients in remission showed insecurity and paranoid ideation. The neuroticism score of CD patients was found to be higher than that of the healthy controls, while that of UC patients was not (94). Another study also showed that CD patients with depression were more likely to deteriorate than UC patients with depression (95). In addition, a prospective study found that depression increased the risk of CD, rather than UC, in women (96). A study in Switzerland recruited 468 adults with CD, who were followed-up for 18 months. The results of the study showed that, among those who were under perceived stress, patients with anxiety and depression were more likely to develop worsening of the disease, indicating the significance of emotional elements (97).

Several recent studies have identified factors beyond disease activity associated with pain burden at birth. Family stressors, such as divorce and loss of family members, were found to increase pain-related distress in children by influencing coping and depression symptoms (98). Thirteen percent of children with CD still suffer from abdominal pain despite clinical remission (99).

Constraint stress has been reported to aggravate spontaneous colitis in *IL-10*^−/−^ mice compared to those without stress (100). Another study demonstrated that neonatal maternal separation induces disruption of the colon barrier and exacerbates colitis symptoms in adult *IL-10*^−/−^ mice (83). Moreover, a recent study revealed that chronic stress damages the gut microbiota and increases susceptibility to DSS-induced colitis in mice. The downregulated expression of mucin-2 and lysozyme caused by stress is implicated in the disturbance of the microbiota (47). Additionally, 12 weeks of WAS significantly increased the relative abundance of the *Clostridium* genus, which produces the toxin phospholipase C in C57BL/6 mice. WAS has also been proven to change the concentration of luminal secreted immunoglobulin A, which is probably connected to gut microbiota alterations and colitis-associated deterioration (51).

#### Effect of Stress on IBD Prognosis

Psychosocial dysfunction has a negative effect on the treatment of IBD in adults. Psychosocial dysfunction causes adverse effects on the quality of life in IBD patients. IBD patients with psychological comorbidities seem to have more hospitalizations than those without (101). The Inflammatory Bowel Disease Questionnaire (IBDQ), which involves bowel symptoms (bowel movements and abdominal pain), systemic symptoms, and emotional and social factors, was applied to assess the health-related quality of life (HRQOL) of IBD patients. The data showed that an increased level of perceived stress, as judged by a 10-item Perceived Stress Scale, is one of the most predictive factors of reduced HRQOL (102). It has been confirmed that psychological symptoms, perceived stress, and disease severity can have a deleterious effect on HRQOL (103, 104). An IBD cohort study in Boston, including 5,405 CD and 5,429 UC patients, found that CD patients with emotional or anxiety disorders had a 28% increase in the risk of surgery compared to those without psychosocial disorders (105).

A recent study found that stress can inhibit endogenous opioids and can switch their signaling in dorsal root ganglion neurons from inhibition to excitation during chronic colitis, causing exacerbated pain and requiring increased doses of opioid analgesics in IBD patients (106). For adolescent patients with IBD, medication non-adherence is regarded as a major health care problem. A systematic review suggested that psychosocial factors, including poor child-coping strategies, family dysfunction, anxiety, and depressive symptoms, are relevant to medication non-adherence, which may lead to an unnecessary escalation in treatment and can jeopardize IBD therapy outcomes (107).

Due to the bidirectional effect between stress and IBD, patients may fall into a vicious cycle, leading to a poor prognosis. Therefore, attention should be paid to the role of stress therapy in the management of IBD.

## Management of IBD: Targeting Stress

According to the present clinical practice guidelines for IBD, therapeutic interventions mainly involve 5-aminosalicylic acid (ASA), corticosteroids, immunomodulators, antibiotics, probiotics, and anti-TNF agents. These traditional treatments can effectively relieve symptoms and promote mucosal healing (108). However, with the in-depth study of the adverse effects of stress on IBD, mental healing seems to be the ultimate treatment goal of IBD and is expected to surpass mucosal healing. Relieving psychological stress is of great benefit to improving symptoms and increasing quality of life. The emerging field of psychogastroenterology focuses on the application of brain-gut psychotherapies, which are considered to be an integral part of the management of digestive diseases (109). Although psychotherapy has limitations, it should be considered to be one of the therapeutic strategies for IBD.

### Pharmacological Treatments

Pain symptoms in IBD patients appear to be associated with inflammation of enteric neurons (110). Enteric neurological abnormalities, including glial cell hyperplasia, and hypertrophy, were more pronounced in CD patients than UC patients (111), and appear to be associated with worsening symptoms and prognosis in CD patients. Intestinal inflammation may induce visceral hypersensitivity through the peripheral and central systems (112). Antidepressants are beneficial for relieving chronic pain, especially in patients with emotional disorders (113). Studies have demonstrated that a high percentage of IBD patients are treated with psychotropic drugs (114), with ~30% of patients taking antidepressants (115). Selective serotonin reuptake inhibitors (SSRIs) and tricyclic antidepressants (TCAs) are the most common drugs used for the treatment of IBD patients with emotional complications, especially anxiety and depression (116). TCAs has been proven to have anti-inflammatory effects in animal intestines. Meanwhile, TCAs seem to relieve severe pain in IBD patients, even at low doses. However, TCAs can also cause side effects, such as dry mouth, blurred vision, and constipation, especially at high doses. These side effects usually recede after a few weeks. Tetracyclic antidepressants are beneficial for patients affected by sleep disorders and pain, but they have not been trialed in IBD patients (117). Moreover, propranolol, a β1-adrenoreceptor/β2-adrenoreceptor inhibitor, has been proven to suppress neutrophil infiltration in the colon and to attenuate tissue injuries caused by chronic stress, suggesting a potential therapeutic value of neuroprotectants, which guard against the recurrence of IBD by inhibiting immune activation (29).

### Psychological Interventions

Nondrug psychological interventions for IBD include cognitive behavioral therapy (CBT), medical hypnosis, and mindfulness meditation. Some studies have shown that these methods reduce gastrointestinal symptoms in IBD patients (117, 118).

IBD-specific CBT is helpful in promoting a higher quality of life and reducing anxiety and depression in IBD patients who have a low level of HRQOL (119). In addition, a benchmark study found that CBT may help IBD patients with moderate to severe mood disorders (120). *Moser G* used gut-directed hypnotherapy (GHT) in the treatment of IBD. The results showed that GHT may prolong the remission duration of patients with inactive UC (121). Clinical hypnosis has been used to guide adolescents to cope with various diseases. Hypnosis can effectively relieve chronic abdominal pain in adolescents with IBD (122). Current mindfulness therapies include mindfulness stress reduction and mindfulness behavioral cognitive therapy, most of which are used in adults and show effectiveness in patients suffering from IBD. Furthermore, the physical and mental intervention of the Breath-Body-Mind Workshop (BBMW) were found to be beneficial for IBD patients for the alleviation of symptoms and emotional disorders (123). As an accepted decompression method, yoga appears to be a safe and efficacious method for the treatment of UC patients (124). In a study of adolescent IBD patients, yoga was found to be an effective complementary therapy. Unfortunately, this was a short survey with a small sample size (31).

A randomized controlled trial showed that psychotherapy (psychoeducation, problem-solving, and relaxation) for patients with IBD did not inhibit disease progression or relapse but enhanced the quality of life (125). A parallel group, randomized and controlled trial evaluated the effectiveness of a disease-specific CBT protocol on anxiety, depressive symptoms and HRQOL in adolescents and young adults with IBD. The preliminary results showed that IBD-specific CBT added to standard medical care did not perform better than standard medical care alone in improving psychological symptoms or HRQOL in youths with IBD (126).

### Brain-Gut-Microbiota Axis Interventions

With the clear evidence of gut dysbiosis in IBD, novel treatments will doubtless require a microbiota-modulating approach (127). This has been an active field of research, with mixed results.

Although exposure to antibiotics is considered to be a potential risk factor for IBD (11, 128, 129), several meta-analyses have revealed that antibiotics are effective in inducing remission and treating flares in patients with IBD (130, 131). Antibiotic therapy remains controversial, especially considering the current mixed results and the potential risks of systemic adverse events and bacterial antibiotic resistance (132, 133). Rifaximin, a non-systemic bactericidal antibiotic, may be therapeutically beneficial for IBD (134). A study found that *Lactobacillus* species were significantly enriched after oral administration of rifaximin. Moreover, rifaximin treatment protected against the intestinal inflammation, barrier damage, and visceral hypersensitivity caused by chronic water avoidance and repeat restraint stressors in Wistar rats (135).

Supplementation with prebiotics and probiotics is favorable for reducing stress-related behavior and HPA activation. Probiotics such as *Bifidobacterium* and *Lactobacillus* can alleviate anxiety and depression (136). Arase et al. studied microbiota-targeted therapies and found that a probiotic *Lactobacillus strain* can assist in protecting against enteritis aggravated by stress (137). Animal studies have found that *Bifidobacterium P122, Lactobacillus LA804*, and *Lactobacillus Switzerland* are beneficial in colitis (138). *B. longum 536* alleviates the symptoms of patients with mild to moderately active UC (139). However, a recent study found that the *B. breve* strain in Yakult did not delay relapse time, compared to a matched placebo in patients with inactive UC. This result may be related to a deficiency in the amount of *B. breve* (140). Most studies have suggested that probiotics are beneficial for IBD patients. The effectiveness and safety of probiotics for alleviating intestinal inflammation in patients with IBD needs more exploration (141).

FMT has become a helpful and increasingly available therapy because of stool banks (142). Randomized controlled studies have indicated that FMT appears to be somewhat effective in the treatment of UC (143, 144). In a prospective trial, 21 children with a median age of 12 years, with IBD refractory to medical treatment, were subjected to a single FMT. Clinical responses were observed in 57 and 28% of the patients at 1 and 6 months after FMT, respectively (145). Paramsothy et al. performed a systematic review and meta-analysis to assess the effectiveness and safety of FMT in IBD. A total of 53 studies (41 in UC, 11 in CD, 4 in pouchitis) published before January 2017 were included. The results showed that the rates of clinical remission in UC, CD, and pouchitis were 36, 50.5, and 21.5%, respectively. Sub-analyses suggested that remission in UC improved with lower gastrointestinal tract administration and an increased number of FMT infusions (146). However, some researchers did not find significant differences in the efficacy of FMT, which may be related to the number and cycle of enemas, preparation process, and limited numbers (147, 148). More large-scale studies evaluating the safety and efficacy of FMT for IBD patients would be a further promising direction.

Rooks et al. demonstrated that genetic inactivation of quorum-sensing *Escherichia coli* regulator C (QseC) can reduce the virulence and colonization capacity in a pathogenic, IBD-associated *E. coli* strain. Further results indicated that biochemical inhibition of QseC can reduce intestinal inflammation in a variety of preclinical IBD models, and provides a new approach for the treatment of colitis (149). Additionally, dietary interventions that modulate the interaction between the immune system and microbiota can also be an option for the treatment of IBD (150).

Studies of the efficacies of psychotherapy and psychopharmacological treatments in patients with IBD are controversial and limited. A systematic literature review has shown that 1/3 of the included 43 studies supported the effectiveness of psychotherapy on the quality of life and disease activity (113). More research is needed to validate this result. As an adjuvant therapy, stress management cannot completely replace drugs. In addition, the study of the gut microbiome and dietary therapy may be future directions for the treatment of IBD.

## Perspectives

Sufficient evidence has indicated that the incidence of psychiatric comorbidities in patients with IBD is higher than that in healthy controls. In turn, these comorbidities exacerbate IBD symptoms and promote intestinal inflammation. The underlying mechanisms may involve alterations of the neuroendocrine system and the brain-gut-microbiota axis. However, the exact mechanisms underlying mucosal immune activation remain to be explored. The effects of psychological stress on IBD should be emphasized, especially in children and adolescents, because of the unique psychological problems encountered in the pediatric population. Thus far, some clinical data support the view that stress management, such as through relaxation exercises, is beneficial to IBD patients, particularly those who are refractory. The psychological score may act as a new measure to evaluate the severity and prognosis of IBD. In the course of future IBD treatment, emotional management, stress release, the use of psychotropic drugs, and care from family needs to be emphasized. Additionally, it should be highlighted that the combination of mucosal and psychological healing as the ultimate goal of therapy will improve the prognosis. The management of IBD goes far beyond traditional drugs and surgical treatment. What is more important is the practice of psychogastroenterology, which appears to be promising.

## Author Contributions

YS and LL wrote the manuscript. HC, RX, KJ, and BW designed the review. YS, LL, and RX participated in the literature search. YS and RX designed and created the figure. HC made critical revisions. All authors read the manuscript and ultimately approved the article.

### Conflict of Interest

The authors declare that the research was conducted in the absence of any commercial or financial relationships that could be construed as a potential conflict of interest.
